# Genotoxic Anti-Cancer Agents and Their Relationship to DNA Damage, Mitosis, and Checkpoint Adaptation in Proliferating Cancer Cells

**DOI:** 10.3390/ijms15033403

**Published:** 2014-02-25

**Authors:** Lucy H. Swift, Roy M. Golsteyn

**Affiliations:** Cancer Cell Laboratory, Department of Biological Sciences, 4401 University Dr, University of Lethbridge, Lethbridge, AB T1K 3M4, Canada; E-Mail: l.swift@uleth.ca

**Keywords:** Cdk1, mitosis, tissue culture, anti-cancer drugs, DNA repair

## Abstract

When a human cell detects damaged DNA, it initiates the DNA damage response (DDR) that permits it to repair the damage and avoid transmitting it to daughter cells. Despite this response, changes to the genome occur and some cells, such as proliferating cancer cells, are prone to genome instability. The cellular processes that lead to genomic changes after a genotoxic event are not well understood. Our research focuses on the relationship between genotoxic cancer drugs and checkpoint adaptation, which is the process of mitosis with damaged DNA. We examine the types of DNA damage induced by widely used cancer drugs and describe their effects upon proliferating cancer cells. There is evidence that cell death caused by genotoxic cancer drugs in some cases includes exiting a DNA damage cell cycle arrest and entry into mitosis. Furthermore, some cells are able to survive this process at a time when the genome is most susceptible to change or rearrangement. Checkpoint adaptation is poorly characterised in human cells; we predict that increasing our understanding of this pathway may help to understand genomic instability in cancer cells and provide insight into methods to improve the efficacy of current cancer therapies.

## 1. Introduction

The human genome is composed of deoxyribonucleic acid (DNA), which is the heritable macromolecule that carries the information essential for life. As a chemical, DNA is susceptible to changes that affect its capacity to perform this role. Cells use highly regulated biochemical pathways to replicate DNA, detect if it is modified, and repair modifications as they arise. Many processes are required to prevent change and to transfer the genome to daughter cells: replication must be accurate, chromosomes must be distributed correctly during cell division, and damage to DNA must be detected and repaired [1]. The fate of a cell, be it healthy or cancerous, is dependent upon the integrity of the genome and its ability to maintain this integrity. By preventing change to DNA, healthy cells ensure their viability and the delivery of a copy of their genetic material to the next generation.

## 2. Types of DNA Damage

The genomic integrity of cells is often challenged by both chemical and physical agents that can modify the bases of nucleotides and modify or break the sugar-phosphate backbone [2]. Agents that damage DNA can be classified in several different ways: they can be endogenous (reactive by-products from processes such as metabolism or inflammation [3]), exogenous (agents present in food, water, or the air [1]) and they can be physical such as ultraviolet (UV) light and ionising radiation or chemicals such as reactive oxygen species (ROS), intercalating agents, alkylating agents and base analogues. In addition to this, agents can directly or indirectly damage DNA, or both. Ionising radiation directly breaks the DNA backbone, but it also produces reactive oxygen species (ROS) that damage DNA in multiple ways. The type of DNA damage has an impact on the fate of a cell by either causing cell death or by being mutagenic, which can lead to diseases such as cancer. In addition to this, DNA damaging agents are commonly used to treat cancer, and understanding how cells respond to them is key to increasing their efficacy.

**Figure 1.**
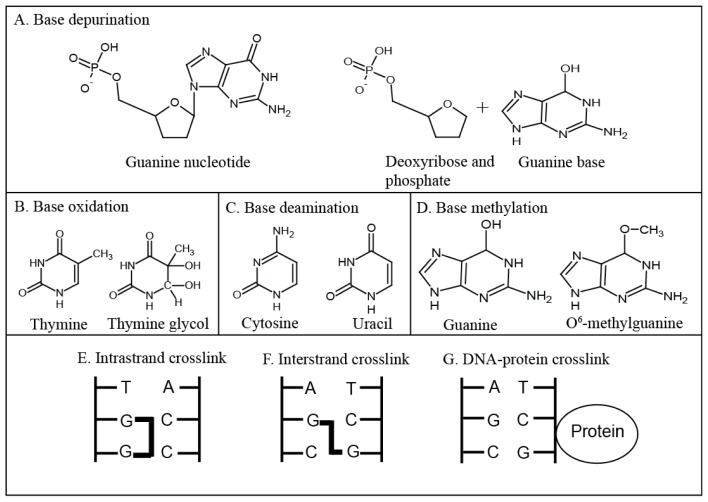
Types of DNA damage. (**A**) Depurination of a guanine nucleotide, creating an abasic site; (**B**) Oxidation of thymine to thymine glycol; (**C**) Deamination of cytosine to uracil; (**D**) Methylation of guanine to *O*^6^-methylguanine; (**E**) An intrastrand guanine-guanine crosslink; (**F**) An interstrand guanine-guanine crosslink; (**G**) A DNA-protein crosslink.

DNA base depurination (Figure 1A) describes the hydrolysis of the bond between a purine and the deoxyribose sugar of DNA, which creates an abasic site [4]. Depurination is a mutagenic process which, if left unrepaired, causes mismatches during DNA replication. Eventually, this process can lead to carcinogenesis [5] as shown in mouse skin papilloma models where it was observed that if guanine was targeted for depurination the mutations observed in the tumour occurred at guanines and if adenine was targeted for depurination the observed mutations in the tumour were at adenines [6].

DNA bases can be oxidised by ROS, causing dozens of major DNA base changes such as thymine glycol [7] (Figure 1B) and 5-hydroxymethyl-2′-deoxyuridine (hmdUrd) [8]. The majority of the DNA base products produced by oxidation cause mismatches during DNA replication, leading to mutagenesis [9]. Some oxidative DNA damage, such as hmdUrd, can cause deletions that have been linked to their processing by the base excision repair pathway [8]. The addition of hydroxyl groups on C5 and C6 of thymine creates thymine glycol, the most common DNA lesion induced by the interaction of thymine with ROS [10]. Thymine glycol is non-planar; the C5 methyl protrudes and prevents base stacking above the lesion, blocking the DNA polymerase during DNA replication [10].

DNA base deamination (Figure 1C) describes the removal of amine groups from the bases adenine, guanine or cytosine [4]. Deamination products include uracil, uracil glycol, xanthine and hypoxanthine [11]. Their generation occurs spontaneously and is enhanced by ROS or agents such as nitric oxide [11]. As with oxidation and depurination, deamination is mostly mutagenic and favours guanine:cytosine to adenine:thymine transitions because cytosine is frequently deaminated to uracil [12] (Figure 1C). Deamination of adenine, guanine or cytosine is often mutagenic because the 4-amino group in pyrimidines and 6-amino group in purines donate hydrogens during the formation of Watson-Crick base pairs [11]. When the bases are deaminated at these residues a keto group replaces the amino group which accepts hydrogen bonds in a normal Watson-Crick base pair [11].

Base alkylation or methylation (Figure 1D) occur when methyl (CH_3_) or alkyl groups (C*_n_*H_2_*_n_*_+1_) are added to a base [4]. Base alkylation can be mutagenic or cytotoxic and bases may be alkylated at any *O*– and *N*–atoms, depending on the alkylating agent and whether the DNA is single stranded or double stranded [13]. Alkylation products such as *O*^6^-methylguanine (Figure 1D) are highly mutagenic and cytotoxic because DNA polymerases frequently incorrectly add a thymine to pair with *O*^6^-methylguanine [14]. The basis of *O*^6^-methylguanine cytotoxicity is through mis-pairing with thymine in the opposite strand. The DNA mismatch repair pathway removes the mis-paired thymine but not the damaged base. This leads to futile cycles of DNA repair and replication that eventually cause single-strand breaks (SSBs) and DSBs [15]. For this reason, organisms synthesise the enzyme *O*^6^-methylguanine-DNA methyltransferase (MGMT) specifically to repair *O*^6^-methylguanine [16]. The effect of base alkylation depends on whether the alkylating agent is monofunctional or bifunctional. Monofunctional alkylating agents modify single bases (usually the N7 of the purines adenine and guanine) causing bulky DNA adducts, whereas bifunctional alkylating agents react with two different DNA bases, which leads to crosslinked DNA [17].

DNA adducts are formed by the addition of large molecules (such as the alkyl groups discussed above) to DNA [18]. Similar to base deamination the addition of molecules to DNA frequently occurs at amino groups of bases and interferes with Watson-Crick base pairing [19]. In addition to this, bulky DNA adducts such as C8 guanine lesions, caused by heterocyclic aromatic amines can block the high fidelity DNA polymerases by preventing deoxycytidine triphosphate from entering the active site [20]. This leads either to DNA damage bypass by the translesion synthesis polymerases or DNA strand breaks.

DNA-DNA crosslinks are formed when two DNA bases are covalently linked to each other, such as through bi-functional alkylating agents that have two reactive sites [21]. If the DNA bases are adjacent to each other the crosslinks are intrastrand (Figure 1E) and if the DNA bases are on different DNA strands the crosslinks are interstrand (Figure 1F). Interstrand crosslinks block the replication machinery by preventing strand separation [22] and bending DNA [23]. They are extremely toxic because they affect both strands, leading to loss of template information [23]. Blocking the replication machinery or DNA repair at interstrand crosslinks can lead to double-strand breaks (DSBs) [21]. DSBs can be cytotoxic but can also lead to insertions, deletions and chromosomal rearrangements if incorrectly repaired. In one study, AS52 Chinese hamster ovary cells were treated with the bifunctional agent chlorambucil or a monofunctional chlorambucil analogue. By using polymerase chain reaction assays and DNA sequencing the monofunctional analogue was shown to induce point mutations whereas the bifunctional chlorambucil induced major genetic deletions [24]. Intrastrand crosslinks can also block the DNA replication fork by bending the DNA double helix [23].

DNA can also be damaged by the covalent crosslinking of proteins to DNA (Figure 1G) [25]. In mammalian cells DNA-protein crosslinks were first shown to occur in fractionated murine lymphoma cells exposed to UV light [26]. The biological effects of DNA-protein crosslinks have been less well studied than other forms of DNA damage because their detection is difficult [27]. It is thought they form bulky helix-distorting adducts that block the DNA replication fork machinery, potentially leading to DNA strand breaks and mutagenesis [25]. DNA is also damaged when nucleic acid-processing enzymes (such as topoisomerases), which usually transiently bind to DNA, are crosslinked to it [28]. These enzymes generate a covalent intermediate with DNA, before being cleaved from it to release a final product [28]. When these proteins are more stably bound to DNA, a covalent or cleavage complex is formed, and these complexes can be converted to SSBs or DSBs when they are met by the transcription or replication machinery [28].

Blocking the replication machinery by base damage or SSBs leads to replication fork stalling and fork arrest/collapse that can form DSBs [18]. DSBs are the most cytotoxic type of lesion, as demonstrated by Biedermann *et al.* (1991) who showed that severe combined immunodeficient mice, deficient in DSB repair, were hypersensitive to direct DSB inducing ionising radiation or bleomycin treatment but normally sensitive to mitomycin C and UV light, which cause DNA inter- and intra-strand crosslinks [29]. It is proposed DSBs are cytotoxic because they are the most difficult type of damage to repair [30]. If both DNA strands are broken they must be re-joined, often without a template, which can lead to insertions, deletions or chromosomal rearrangements [31]. In an effort to maintain genomic stability, cells have evolved elaborate pathways to arrest the cell cycle in response to damaged DNA and DSBs.

## 3. DNA Damaging Agents as Anti-Cancer Drugs

Cancer is a complex disease characterised by at least six hallmark characteristics [32]. Some of these hallmarks, such as proliferation and resistance to cell death (including apoptosis) act at a cellular level and are frequently caused by changes in the genome. An increased rate of cellular proliferation is frequent, but not exclusively found in cancer cells [32]. Most cancer cells divide more often than normal cells and the process of cell division can be targeted to treat cancer patients. The aim of targeting cell proliferation is to arrest the cell cycle and/or cause cancer cell death using cytotoxic compounds (chemotherapy) or ionising radiation (radiation therapy). DNA is one of the main targets of these therapies because DNA replication is an essential phase of the cell cycle. Many of the cytotoxic agents commonly used to treat cancer patients cause high levels of DNA damage, that initiate cell cycle checkpoints, leading to cell cycle arrest and/or cell death [17]. As discussed below, DNA can be damaged by cytotoxic agents that have different mechanisms of action and cause the types of DNA damage previously discussed. A summary of these cytotoxic agents, their mechanism of action and the type of DNA damage that they cause is provided in Table 1.

### 3.1. Alkylating Agents

**Figure 2.**
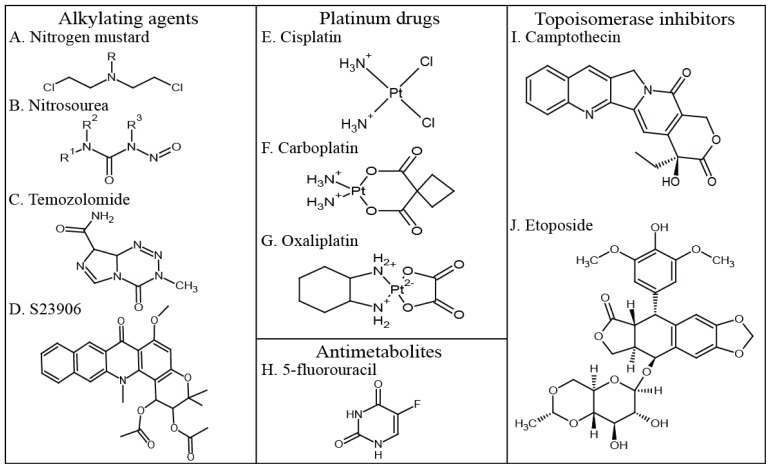
Structures of DNA damaging agents. (**A**) The general structure of a nitrogen mustard alkylating agent; (**B**) The general structure of a nitrosourea alkylating agent; (**C**) Temozolomide, a methylating agent; (**D**) S23906, an atypical alkylating agent; (**E**) The platinum drug cisplatin; (**F**) The platinum drug carboplatin; (**G**) The platinum drug oxaliplatin; (**H**) The antimetabolite 5-fluorouracil; (**I**) Camptothecin, a topoisomerase I inhibitor; (**J**) Etoposide, a topoisomerase II inhibitor.

Alkylating agents are electrophiles that covalently transfer alkyl-groups onto the DNA bases [17]. There are several different groups of alkylating agents: the nitrogen mustards (Figure 2A), nitrosoureas (Figure 2B), aziridine compounds, alkyl sulphonates and the triazine compounds [33]. The nitrogen mustards and nitrosoureas were the first DNA damaging drugs used to treat cancer patients [34,35]. They were developed from the study of soldiers exposed to sulphur mustard gas in World War I [36] and in World War II, when an American ship containing mustard-gas bombs was sunk in Italy [34]. It was observed that the sulphur mustard gas caused bone marrow suppression and a decrease in the amount of lymphoid tissue [37]. Subsequently nitrogen mustard, a compound related to sulphur mustard was used to treat a patient with non-Hodgkin’s lymphoma, causing a temporary remission of the tumour and establishing that drugs could be used to cause tumour regression [36,38].

The nitrogen mustards are bifunctional alkylating agents that damage DNA by forming guanine-guanine and guanine-adenine interstrand crosslinks. Mechlorethamine, bendamustine, melphalan, chlorambucil, ifosfamide and cyclophosphamide are the nitrogen mustards most commonly used today [33]. Closely related to the nitrogen mustards are the aziridine compounds, such as mitomycin C and thiotepa, which also form guanine-guanine and guanine-adenine interstrand crosslinks [33,39]. Busulfan is the main alkyl sulphonate currently in use [33] and is a bifunctional alkylating agent that produces guanine-guanine intra- or inter- strand crosslinks [40]. The majority of nitrosoureas are monofunctional chloroethylating agents that add chloroethyl groups to the N7 and O6 of guanine [39]. Examples of chloroethylating agents are carmustine (BCNU, Figure 4), lomustine (CCNU) and nimustine (ACNU) [33]. *O*^6^-chloroethylguanine adducts are particularly cytotoxic because they react with cytosine to generate guanine-cytosine interstrand crosslinks [39]. Finally, the triazine compounds such as dacarbazine, procarbazine and temozolomide (Figure 2C) are monofunctional methylating agents whose cytotoxicity is linked to their production of *O*^6^-methylguanine, despite this accounting for only approximately 5% of DNA adducts generated by these compounds [39,41].

In recent years the use of temozolomide (TMZ) (Figure 2C) in conjunction with radiotherapy has become standard in the treatment of glioblastoma [42] because of its success in crossing the blood-brain barrier [43]. This is significant because few drugs are licensed to treat glioblastoma [43], an aggressive type of brain tumour associated with poor prognosis [44]. Since the introduction of TMZ in 2005, in the United States median patient survival has increased by several months [44]. In addition to patient benefit, TMZ has a low toxicity profile, increasing a patient’s quality of life and is orally administered [43]. The mean peak plasma concentration of TMZ calculated from patients treated with 200 mg/m^2^/d for 5 days was measured on day 5 as 104 μM, as shown in Table 2 [45].

A more recently discovered and atypical monofunctional alkylating agent is S23906 [46] (Figure 2D). S23906 is related to the natural pyranoacridone alkaloid acronycine, which was first identified in extracts from the plant *Sarcomelicope simplicifolia* [47]. Chemical modification of acronycine derivatives led to the discovery of the potent alkylating agent S23906, which displayed anti-cancer activity in preclinical models of solid tumours. Characterisation of the mechanism of action of S23906 revealed that it alkylated the N2 of guanine in the minor groove of DNA [46,48]. In addition to modifying this site, an activity found in a few of the alkylating agents such the marine alkaloid ecteinascidin 743, S23906 had the unique property of inducing DNA helix opening. The effects of S23906 upon cells treated with pharmacological concentrations are only detected during *S*-phase, and are blocked by the addition of DNA polymerase inhibitors [46,49]. Removal of S23906 from culture media after treatment does not change the cytotoxic effects when compared to continuous treatment, suggesting that cells are unable to repair S23906 adducts effectively. The unusual mechanism of S23906 modification of DNA provides an opportunity to examine the cellular response to damaged DNA and its relationship to cell death [46,48].

### 3.2. Platinum Drugs

*Cis*-diamminedichloroplatinum(II) (cisplatin) (Figure 2E) and its analogues carboplatin (Figure 2F) and oxaliplatin (Figure 2G) are widely used to treat testicular or ovarian cancers [17,60]. Cisplatin is a platinum based inorganic molecule [61] that has a mechanism of action similar to that of alkylating agents [60]. It forms covalent bonds between DNA (commonly the N7 of the purine bases) and the platinum moiety, making either monofunctional adducts or intra- and inter- strand and DNA-protein crosslinks [35,60]. The most common type of DNA damage is intrastrand crosslinks between adjacent guanines. The various adducts and crosslinks caused by cisplatin have a multitude of effects on a cell including DNA unwinding, DNA bending and impeding DNA replication and transcription, which can lead to DNA strand breaks [60], but it is not *S*-phase dependent.

Despite being effective and widely used, cisplatin causes severe side-effects such as nephrotoxicity and is associated with resistance, especially in ovarian cancer [60,62]. This resistance may be acquired during therapy or be intrinsic to the tumour and may result from increased tolerance and/or repair of the DNA damage, increased detoxification of cisplatin in the cytoplasm and decreased membrane transport into the cell [60,62]. To overcome some of the resistance and side effects associated with cisplatin, analogues such as carboplatin and oxaliplatin were identified and introduced [60]. Carboplatin and oxaliplatin have the same mechanism of action as cisplatin but have different pharmacological properties, which reduce the many severe side effects associated with cisplatin [35,60]. The peak plasma concentration of total platinum detected post-treatment with carboplatin ranges from 70–130 μM [53,54,63], while the peak plasma concentration of total platinum following treatment with oxaliplatin has been reported as 10 μM, as shown in Table 2 [55]. The therapeutic range of cisplatin in the blood is reported as being 3–16 μM [50], although studies have reported peak plasma concentrations of total platinum ranging from 12–40 μM post-intravenous treatment with 100 mg/m^2^ cisplatin (Table 2) [51,52,63]. Interestingly low levels of platinum can be detected in the blood up to three months after a patient’s last treatment [50].

### 3.3. Antimetabolites

Antimetabolites used in cancer therapies typically have chemical structure that are related to nucleotide metabolites [17] and either inhibit biosynthetic processes or are incorporated into nucleic acids such as DNA and RNA [64]. Inhibiting nucleotide metabolism pathways depletes cells of deoxynucleotide triphosphates (dNTPs), preventing DNA replication [17]. In contrast, incorporation of antimetabolites into DNA stalls or blocks DNA replication [17]. The fluoropyrimidine 5-fluorouracil (5-FU) (Figure 2H) is an antimetabolite commonly used to treat colorectal cancers [64]. 5-FU can be incorporated into RNA and DNA in the place of uracil or thymine [65] and also inhibits thymidylate synthase, the enzyme that synthesises deoxythymidine monophosphate from deoxyuridine monophosphate [64]. Inhibition of this pathway depletes the pool of all nucleotides, causing severe DNA damage [64,66,67]. In addition to the incorporation of 5-FU into DNA, repair by the nucleotide excision repair pathway results in further 5-FU incorporation, eventually leading to DNA strand breaks through futile cycles of 5-FU misincorporation, excision and attempted repair [64]. 5-FU has a short half-life of 10–15 min and patients are therefore treated with 5-FU for a prolonged time [56]. The peak plasma concentration of 5-FU has been shown to be highly variable both in the same patient during a treatment course and between patients [56]. When patients were treated intravenously with 1750 mg/m^2^/d for 72 h, the mean peak concentration was 10 μM (Table 2) [56].

### 3.4. Topoisomerase Inhibitors

Topoisomerases are essential enzymes that relax DNA supercoiling during DNA replication and transcription, by introducing transient nicks to relieve torsional stress [68]. There are two types of topoisomerases, type I and type II, and anti-cancer drugs that target both types are widely used to treat patients. Type I topoisomerases (TOP1) break one DNA strand and pass the uncut strand through this break, whereas type II topoisomerases (TOP2) break both strands of DNA to unwind it [65]. The strand cleavage action of topoisomerases avoids causing genomic change by covalently attaching to the DNA, forming enzyme-DNA cleavage complexes [69]. Topoisomerase inhibitors act by transiently trapping the enzymes in these intermediate complexes, often preventing the nicks from re-ligating and leading to DNA strand breaks [68,69]. TOP2 inhibitors cause DSBs directly whereas TOP1 inhibitors first cause SSBs, which are converted to DSBs when they are met by a replication fork [69,70]. The ends of TOP1 nicks created by the TOP1 inhibitors have a 5′-hydroxyl and 3′-phosphate-protein intermediate [71], which is a property that may be exploited for cancer therapy.

Camptothecin (CPT) (Figure 2I) is a TOP1 inhibitor [72] and its water soluble derivatives topotecan and irinotecan [68] are widely used to treat colorectal, ovarian and lung cancer. CPT binds to DNA-TOP1 cleavage complexes, blocking re-ligation and resulting in the accumulation of transient SSBs [68,72]. Camptothecins rapidly diffuse from TOP1 cleavage complexes reversing the inhibition of TOP1, but if this inhibition is maintained then the SSBs are converted to DSBs when they are encountered by the replication fork [68,70]. The peak plasma concentration of 9-nitro camptothecin is reported as being highly variable, with a maximum concentration of 75 nM detected 3.5 h post-treatment [57].

Two classes of drugs that target TOP2 exist, the TOP2 poisons and the TOP2 catalytic inhibitors [73]. Although the precise mechanism of action of the TOP2 poisons remains unknown [73], it is similar to that of CPT; they transiently bind to the TOP2 cleavage complexes and prevent re-ligation of the DNA strand breaks [69,73]. The TOP2 catalytic inhibitors appear to inhibit the enzymatic activity of TOP2, as opposed to binding to the TOP2-DNA cleavage complexes [73]. Etoposide (Figure 2J) is a TOP2 poison [74] used to treat a range of cancers [75], most commonly small cell lung and testicular cancer. A negative aspect of treatment with TOP2 inhibitors is that their use is linked to the occurrence of secondary leukaemia and other malignancies [73,76,77]. The peak plasma concentration of etoposide can also be highly variable and in addition to this etoposide can be given at variable doses by different methods, for example intravenously or orally, depending on the cancer being treated [58,59]. In ovarian cancer patients given an oral dose of 100 mg/m^2^/d for 8–15 days the peak plasma concentration of etoposide detected on day 1 of treatment was 14 μM [58]. In patients treated intravenously with high doses of 400–800 mg/m^2^/d for 3 days peak plasma concentrations were proportional to the dose of etoposide given and ranged from 45–194 μM [59]. This information is summarised in Table 2.

### 3.5. Ionising Radiation

Ionising radiation is a physical agent that damages DNA and can both cause and treat cancer. Ionising radiation can be used alone or in combination with anti-cancer drugs and it is estimated that approximately 50% of all cancer patients receive ionising radiation at some point during their treatment [78]. Ionising radiation is delivered in fractions to a final dose. Standard fractionation involves single daily doses of 1.8–2.0 Gray/day to a weekly dose of 9.0–10 Gray [79,80]. The final dose a tumour receives is limited by the tolerance of the surrounding tissue [81] and doses typically range from 20–70 Gray depending on the tumour type [82]. Ionising radiation has both a direct and an indirect action on DNA. Its direct action damages the DNA backbone, forming DNA strand breaks [1], by ionising or exciting atoms in DNA [83]. Ionising radiation indirectly damages DNA by producing ROS, atoms or molecules that carry an unpaired electron in their outer shell causing them to be highly reactive [83]. Common ROS are hydrogen peroxide, hydroxyl radicals and super oxide anions [3] all of which are formed as oxygen is reduced to water in the mitochondria. ROS can form covalent bonds with DNA leading to a diverse range of modifications including adduct formation, base oxidation (Figure 1B), base deamination (Figure 1C), formation of abasic sites (Figure 1A) and formation of SSBs and DSBs or DNA-protein crosslinks (Figure 1G) [1]. Mitotic catastrophe is proposed to be the main mechanism of cell death after exposure to ionising radiation [84,85] and the observation of chromosomal aberrations has been associated with its use for many years [83].

## 4. The DNA Damage Response (DDR)—DNA Damage Signalling and Cell Cycle Checkpoints

To prevent the transmission of damaged DNA to daughter cells during cell division, damaged DNA must be repaired. This depends on DNA damage repair pathways as well as cell cycle checkpoint activation to arrest the cell cycle. If DNA damage is irreparable cells may signal for senescence (growth arrest), apoptosis (programmed cell death) [2] or other pathways leading to cell death. Cell cycle checkpoints can be activated at G1, in *S*-phase, at G2/M or in mitosis [2]. The DNA damage response (DDR) is a network of interacting pathways made up of DNA damage sensors, transducers and effectors [86] that initiate checkpoints, signal for DNA repair and then either re-entry into the cell cycle, senescence or cell death, depending on the outcome of DNA repair [87]. The main proteins and pathways of the DDR are conserved from yeast to humans, highlighting the importance of this response in eukaryotic organisms [88].

**Figure 3.**
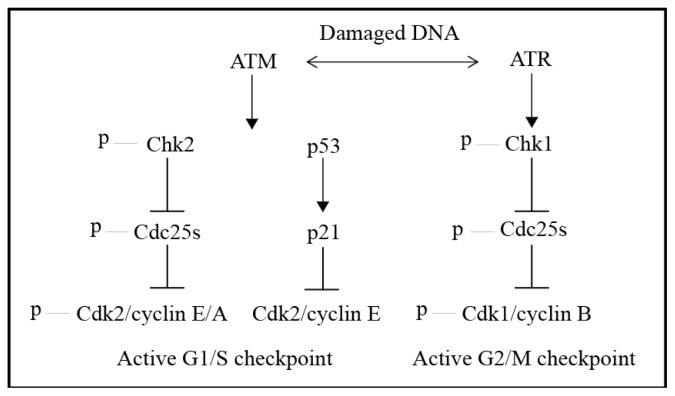
An overview of the DNA damage response (DDR). In response to DNA damage the ataxia telangiectasia mutated (ATM)/ATM and Rad3-related (ATR) kinases are activated and phosphorylate many substrates including proteins involved in checkpoint activation, DNA replication and DNA damage repair. When activated checkpoint kinase 1 (Chk1) and checkpoint kinase 2 (Chk2) inactivate the Cdc25 phosphatases, initiating checkpoints which prevent cells from progressing through the cell cycle by maintaining the inhibitory phosphorylation of the cyclin-dependent kinases (Cdks). ATM is also responsible for activating p53 which activates the G1/S checkpoint through inhibition of Cdk2/cyclin E by p21.

The response to damaged DNA is initiated by the large serine/threonine kinases, ataxia telangiectasia mutated (ATM) and ATM and Rad3-related (ATR) [2,35]. ATM and ATR are known as the signal transducer kinases and are core components of the DDR (Figure 3). The sensors and effectors of the DDR are still being characterised and differ depending on the type of DNA damage that occurs. In response to DNA damage ATM and ATR phosphorylate a multitude of substrates [2] and an extensive proteomics study on cultured cells by Matsuoka *et al.* in 2007 identified more than 900 sites on 700 proteins that were phosphorylated in response to 10 Gray of ionising radiation [89]. The two kinases respond to different types of DNA damage, although there is increasing evidence to suggest that there is interaction and overlap between the pathways [90].

ATM is present in the cell as an inactive homodimer [91] and responds to DSBs [2]. ATM is therefore activated by ionising radiation [90] and genotoxic agents that induce DSBs such as etoposide [73,92] and CPT which can activate ATM and ATR when the SSBs it causes are processed to DSBs. When ATM detects damaged DNA it is activated by auto-phosphorylation, which causes the homodimers to dissociate and form active monomers [91]. These monomers are recruited to DSBs by the Mre11-Rad50-Nbs1 (MRN) complex, a regulator for ATM [93] and ATM is then involved in checkpoint signalling, DNA end processing and DNA recombination or repair [94] by phosphorylating multiple substrates [95] including the histone variant H2AX and checkpoint kinase 2 (Chk2). The histone variant H2AX is phosphorylated on serine 139 (γH2AX), signalling that DNA damage has occurred [96] and is required for the assembly of proteins associated with the DDR and DNA damage repair at DNA damage sites [90]. Chk2 is activated by phosphorylation of threonine 68, which initiates the G1/S phase checkpoint through indirect inhibition of cyclin-dependent kinase 2 (Cdk2) (Figure 3). ATM also activates the p53 response to DNA damage [97] and interacts with ATR [90].

ATR is regarded as the main effector of the G2/M checkpoint [90] and responds to DNA damage such as DNA crosslinks, DNA adducts and DNA breaks [98] because it detects single-stranded DNA (ssDNA) that is present at stalled replication forks or generated by DSB end-processing [86]. ATR is therefore activated by DNA damaging agents such as 5-FU [99], UV light [90], the alkylating agents [100] and the platinum drugs [101] and is also activated by ATM responding to DSBs by DNA end resection [102,103]. ATR is found in a complex with ATR-interacting protein (ATRIP) [104] which interacts with replication protein A (RPA), a protein that coats ssDNA [98,105]. Once recruited by RPA, ATR is further activated by DNA topoisomerase II binding protein 1 (TOPBP1) [106] which is recruited to the ssDNA by the Rad9-Rad1-Hus1 (9-1-1) complex [107]. The major substrate of ATR is checkpoint kinase 1 (Chk1) which is activated by phosphorylation at serine residues 317 and 345 [108,109], present in the regulatory *C*-terminal domain [110]. In addition to phosphorylation by ATR, the Rad17-replication factor C (RFC) complex, the 9-1-1 complex [98] and claspin are necessary for Chk1 activation [111–113]. The Rad17-RFC complex acts a clamp loader at RPA bound ssDNA, engaging the 9-1-1 complex [98,114] whereas claspin is an adaptor protein [111] that links ATR and Chk1, allowing ATR to phosphorylate Chk1 at serines 317 and 345 [115].

Once activated Chk1 prevents the activation of cyclin-dependent kinase 1 (Cdk1) which in complex with cyclin B is responsible for mitotic entry. Cdk1 is inhibited by phosphorylation of threonine 14 and tyrosine 15 by Myt1 and Wee1 respectively. The cdc25 phosphatases remove these inhibitory phosphate groups, activating Cdk1, and entry into mitosis occurs. Activated Chk1 phosphorylates the cdc25 phosphatases, targeting them for sequestration or ubiquitination and degradation [116,117]. In the absence of cdc25 phosphatases, the Cdk1 complex cannot be activated and cells do not enter mitosis (Figure 3). In contrast, when Cdk1 becomes activated it phosphorylates and stimulates many proteins and enzymes responsible for the major steps of mitosis such as nuclear membrane breakdown, microtubule reorganisation, chromatin condensation and changes in the actin cytoskeleton that cause mitotic cells to adopt a rounded morphology [118].

**Figure 4.**
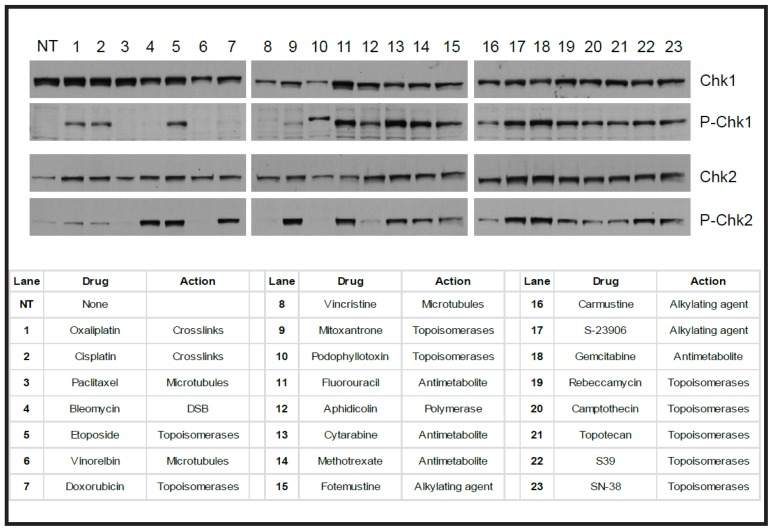
The majority of genotoxic agents activate the DNA damage response (DDR). Human cancer cells were treated with 1 of 23 cytotoxic agents, of which 19 were from the genotoxic category. Cell extracts were analysed by western blotting with antibodies directed to either phos-S345 Chk1 or phos-T68 Chk2, which are the activated forms of Chk1 or Chk2 in response to damaged DNA (see Figure 3). The presence of total Chk1 or total Chk2 was confirmed in each sample. In each case where cells were treated with a genotoxic agent, including the hemisynthetic agent with an unusual mechanism of action, S23906, the DDR pathway was activated. In cases where non-genotoxic agents were used, such as lanes 3, 6, or 8, the DDR pathway was not activated. NT refers to cells that were not treated. The list of cytotoxic agents and the lane number is provided.

Activation of cell cycle checkpoints by the DDR provides cells with time to repair damaged DNA and allows them to either proceed with the cell cycle (with damaged or repaired DNA), senesce or die. One of the striking features of the DNA damage pathway is its capacity to detect many different types of damaged DNA. This capacity is demonstrated in Figure 4, in which HT-29 human colon carcinoma cells were treated with 22 different cytotoxic cancer drugs. Of these, 19 were genotoxic by their mechanism of action. Despite the varied mechanisms of damaging DNA, cells were able to activate either Chk1 or Chk2. In the presence of the non-genotoxic microtubule poisons, paclitaxel and vinorelbine, neither Chk1 nor Chk2 were activated. Although progress has been made in identifying the pathways that enable either of these outcomes, it is unclear how cells with damaged DNA determine if they will either survive or die [35].

In non-cancerous cells the G1/S checkpoint is an important checkpoint initiated by the ATM-Chk2-p53-MDM2-p21 pathway [2]. In contrast, most cancer cells have a defective G1 checkpoint because of mutations in the p53 [119] or retinoblastoma tumour suppressor genes or an imbalance in Cdks and cyclins [120]. This means that in many cancer cells the most important checkpoint in response to DNA damage is the G2/M checkpoint associated with the ATR-Chk1-Cdc25 phosphatase pathway, as opposed to the p53 pathway that initiates apoptosis [121].

## 5. Cell Cycle Checkpoints and Checkpoint Adaptation

Recently there has been much interest in discovering and characterising compounds that target the DDR and cell cycle checkpoints to enhance the efficacy of the genotoxic drugs. For example Cdk1 inhibitors can be used to prevent cells from entering mitosis [122,123] and Chk1 inhibitors can be used to prevent cells from engaging a long G2/M arrest [121], to enhance mitotic cell death. However, whereas the pathways involved in the initiation of cell cycle checkpoints during the DDR are relatively well characterised, in comparison the pathway(s) associated with a cell’s outcome after the initiation of a cell cycle checkpoint are less well understood. It is therefore important to understand what happens post-checkpoint initiation, so that anti-cancer drugs targeting the DDR and cell cycle checkpoints can be used more successfully.

After checkpoint initiation a cell may die by apoptosis, necrosis, autophagy or mitotic cell death. The cellular response post-checkpoint initiation may be different depending on: the quantity and type of DNA damage, which tissue a cell originates from, whether a cell is cancerous or not, which checkpoint has been activated, and whether a cell contains mutated genes such as p53 that may affect its ability to signal for a particular death pathway such as apoptosis. For example following radiation treatment apoptosis appears to be p53 and cell type dependent [83]. Lymphoid and haemopoietic cells undergo rapid apoptosis following radiation treatment whereas many tumours composed of proliferative cells undergo a mitosis induced cell death [83]. For a cell to die by mitosis after a DNA damaging event it must have overcome the G2/M checkpoint and two possible ways that this may occur are checkpoint recovery and checkpoint adaptation [87,124]. Checkpoint recovery occurs when cells enter mitosis after repairing damaged DNA [87] whereas checkpoint adaptation occurs when cells enter mitosis with damaged DNA [125].

Mitosis as a mode of cell death after treatment with DNA damaging agents has been recognised for many decades [126]. Little is understood about how cells overcome the G2/M checkpoint to enter mitosis, when they do it and why. In our opinion, entry into mitosis after treatment with genotoxic agents has been overlooked for many years and we propose that checkpoint adaptation is one mechanism by which this mitotic entry occurs. We predict that increasing our understanding of cell fate and the pathways initiated to achieve this fate after a DNA damaging event could be used to enhance current cancer therapies and inform future anti-cancer drug use and discovery.

**Figure 5.**
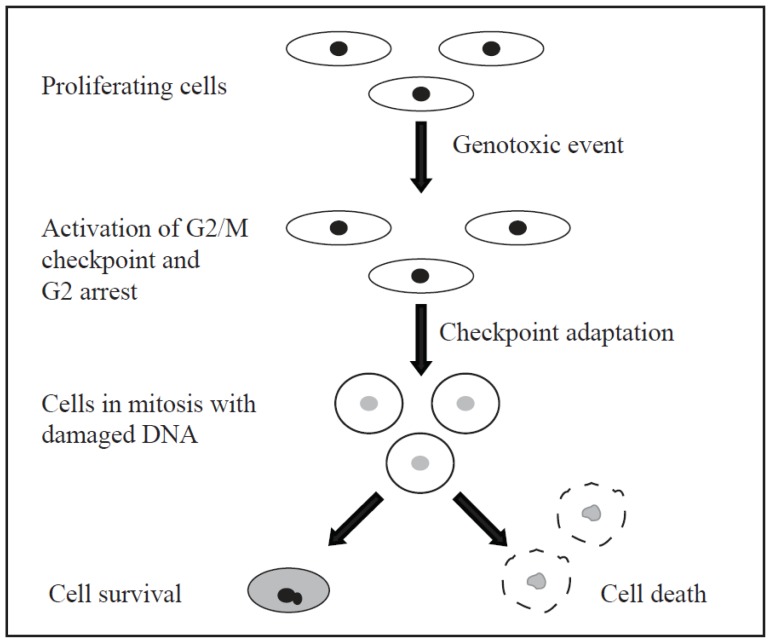
A model of checkpoint adaptation. A genotoxic event damages the DNA of proliferating cell, which leads to the activation of the G2/M checkpoint and arrest in G2. The cells undergo checkpoint adaptation; they enter mitosis with damaged DNA. The majority of cells undergo cell death but some cells may survive, likely with changes to their genome.

Checkpoint adaptation is defined by three sequential steps: (i) a DNA damage induced cell cycle arrest; (ii) overcoming this cell cycle arrest; and (iii) resuming the cell cycle with damaged DNA [125,127]. The steps of checkpoint adaptation are shown in Figure 5. Checkpoint adaptation at the G2/M checkpoint was first described in *Saccharomyces cerevisiae* by Sandell and Zakian (1993) who observed that after induction of DSBs in DNA repair deficient cells, cells responded by initiating a G2/M arrest and then proceeded to divide with damaged DNA [128]. Checkpoint adaptation in yeast has since been reported by several different groups and led to the identification of several possible pathways that have yet to be studied in human cell models [125,129–132].

In 2004, Yoo *et al.* described checkpoint adaptation in *Xenopus* egg extracts that entered mitosis despite a DNA replication block induced by treatment with aphidicolin [129]. This was a surprising result because it was widely thought that checkpoint adaptation would only occur in single-celled organisms, which, unlike multicellular organisms would not risk having different genomes within the same organism if DNA repair was not successful [129,133]. The research by Yoo *et al.* (2004) prompted the question of whether checkpoint adaptation occurred in mammalian cells and in 2006 Syljuasen *et al.* published the first report of checkpoint adaptation in human cells [129,134]. Syljuasen *et al.* (2006) showed that U2OS osteosarcoma cells entered mitosis with damaged DNA induced by ionising radiation [134]. This research was based on the knowledge that human cancer cells can undergo cell division after treatment with ionising radiation, before they die [83]. Following this in 2011, Rezacova *et al.* reported that 26% of MOLT4 lymphocytic leukaemia cells treated with fractionated irradiation initiated a G2/M arrest 48 h after the first treatment, and then entered mitosis with damaged DNA [135]. Most recently Kubara *et al.* (2012) published a cell-based model of checkpoint adaptation which uses HT-29 human colon carcinoma cells [127]. They reported that mitosis is a key cellular response to genotoxic agents in human cells, showing that both HT-29 and M059K glioma cells enter into mitosis with damaged DNA induced by pharmaceutically relevant concentrations of the TOP1 inhibitor CPT [127].

The biochemical pathways that regulate checkpoint adaptation in human cells are not yet well understood. However it has been shown that after treatment with the genotoxic agent CPT 90% of cells enter into mitosis with damaged DNA and up to 98% of these cells will die, while 2% may survive [127]. It is therefore hypothesised that checkpoint adaptation may be a key cellular response to irreparable DNA damage, occurring to ensure the majority of damaged cells die by allowing cell death pathways such as apoptosis and necrosis to occur in other phases of the cell cycle [124]. This hypothesis is supported by the knowledge that human cancer cells commonly enter into mitosis before dying after treatment with ionising radiation [83,85] and different genotoxic agents [46,136]. Entry into mitosis, with concurrent activation of Cdk1, may in part explain the long observed relationship between Cdk1 activity and cell death [137,138].

We propose that the relationship between mitosis and damaged DNA in some proliferating cancer cells could be targeted therapeutically to increase the efficacy of current cancer treatments. In these cases, cancer cells might enter mitosis more frequently than normal cells in response to DNA damage because they are more likely to be deficient in different aspects of cell cycle checkpoints [139], with 50% of human cancers containing a defective p53 gene [140,141]. Indeed, it has been shown that inhibiting the checkpoint kinases Chk1 and Chk2 can induce mitosis in treated human cancer cells [142–146].

Studies have shown that mitotic entry occurs before cell death in various cell lines treated with different DNA damaging agents. The diversity of cancer cell lines that have responded to a range of DNA damaging treatments by entering mitosis demonstrates that entry into mitosis with damaged DNA and mitotic cell death should not be overlooked in cancer research. Following treatment with ionising radiation HeLa cervix adenocarcinoma cells enter mitosis [147] and both U2OS and MOLT4 cells undergo checkpoint adaptation [134]. Low dose bleomycin (a radiomimetic drug that induces DSBs) was shown to induce mitotic cell death in DC-3F Chinese hamster lung fibroblasts [148] whereas M059K and M059J glioma cells both entered mitosis post-bleomycin treatment [149]. HT-29 and M059K cells treated with the TOP1 inhibitor CPT undergo checkpoint adaptation [127] whereas HeLa cells treated with the TOP2 inhibitor etoposide were enlarged and micronucleated, suggesting that they had entered mitosis after treatment [150]. U2OS cells treated with etoposide were also shown to enter mitosis, and strikingly 2-colour FISH analysis linked the cells surviving mitosis to a specific 11q23 chromosomal translocation associated with TOP2 inhibitor related secondary leukaemia [76]. After treatment with cisplatin, CHO/UV41 Chinese hamster ovary cells [151] entered mitosis, SKOV-3 ovarian carcinoma cells [152] displayed micronuclei and HT-29 cells undergo checkpoint adaptation (unpublished results). Treatment of different glioma cells with TMZ demonstrated that their response was p53 dependent [143]. The p53 deficient U87-MG-E6 and LN-Z308 cell lines induced a transient G2/M arrest but the p53 proficient cell line U87 induced a long G2/M arrest followed by senescence [143]. Finally S23906 with its unique mechanism of action previously discussed was also shown to induce Chk1 activation, followed by mitotic catastrophe in HeLa and HT-29 cells [46]. This study was important because S23906 causes an atypical form of DNA damage and yet still induced mitotic catastrophe, suggesting that mitotic catastrophe is a major response to damaged DNA.

In addition to these studies Chang *et al.* (1999) treated HT1080 3’SS6 human fibrosarcoma cells with doxorubicin, aphidicolin, cisplatin, γ-irradiation, cytarabine (an antimetabolite) and etoposide and showed that between 45% and 64% were micronucleated as opposed to 1.5% of not treated cells [153]. In the same study Chang *et al.* (1999) also treated 14 different cell lines with moderate doses of doxorubicin as determined for each cell line, and detected micronuclei in 12 of the 14 cell lines with between 20% and >80% of cells containing micronuclei post-treatment, depending on the cell line [153].

Several studies have indicated that cellular response to a treatment may be dose dependent. Studies with 5-FU [154], doxorubicin [155] and bleomycin [148] describe different responses to high and low doses of these treatments. Low-dose treatment of three colon adenocarcinoma cell lines with 5-FU led to mitotic catastrophe, while high-dose treatment induced direct cell death by apoptosis [154]. The same observations were made when DC-3F cells were treated with low and high doses of bleomycin, treatment with a low dose led to mitotic catastrophe and treatment with a high dose led directly to apoptosis [148]. Similarly, low dose treatment of five hepatocellular carcinoma cell lines with doxorubicin (an antibiotic TOP2 inhibitor) induced a senescence like phenotype followed by mitotic catastrophe while high dose treatment induced direct cell death by apoptosis [155]. These studies indicate that the relationship between mitotic cell death, checkpoint adaptation and dose (or concentration) of the genotoxic agent is still not well described. It is therefore worthwhile confirming the concentration of the genotoxic agent used in studies of cell death, including checkpoint adaptation.

Importantly, there is clinical evidence that cells pass through mitosis after acquiring DNA damage by ionising radiation. Several clinical studies used cytological staining and light microscopy and revealed an increased number of micronuclei in oral [156–158] or cervical [159,160] carcinoma samples post-treatment. Micronuclei are formed when lagging acentric chromosome or chromatid fragments or whole chromosomes are not present in daughter cells at the completion of mitosis, instead becoming enclosed in a separate nuclear membrane [161]. Micronuclei are therefore associated with entry into mitosis with damaged DNA. In some cases micronuclei are also associated with the term mitotic catastrophe [85,162–164]; however there is a lack of consensus regarding the exact definition of mitotic catastrophe, discussed below.

Mitotic catastrophe has been defined as cell death caused by aberrant mitosis associated with spontaneous premature chromosome condensation and multiple micronuclei [136,165], as cell death resulting from inappropriate entry into mitosis [162,165], or as cell death occurring during or shortly after a failed mitosis [165,166]. More recently mitotic catastrophe has been defined as a mechanism that senses mitotic failure and responds to it by inducing either apoptosis, necrosis or senescence leading to three different consequences: (i) cell death during mitosis (mitotic death); (ii) cell death after mitotic exit; and (iii) senescence after mitotic exit [165,167]. It is also debated whether mitotic catastrophe is a form of cell death in its own right [136,164] or whether cells die in mitosis by apoptosis or necrosis [84,139].

One pathway that allows entry into mitosis with damaged DNA is checkpoint adaptation. Many of the cell models studied using genotoxic agents have yet to apply tests of the three steps that define checkpoint adaptation, namely, a DNA damage induced arrest, overcoming this arrest and resuming the cell cycle with damaged DNA. The current model to test for checkpoint adaptation uses HT-29 cells [127]. HT-29 cells are ideal for studying checkpoint adaptation in human cells because they have chromosome instability properties that enable them to display mitotic entry [168]. Furthermore, they are a polarised cell in culture and assume a strikingly rounded morphology when in mitosis, which enables one to collect mitotic cells by mechanical shake-off.

To demonstrate the process of checkpoint adaptation experimentally DNA damage can be detected using immunofluorescence microscopy for histone γH2AX [127,134] and the comet assay (unpublished data). Phosphorylation of the histone variant H2AX on serine 139 (γH2AX) is a sensitive marker for the signalling of DNA damage [169] whereas the comet assay is able to detect the presence of DNA strand breaks in individual cells [170]. Cell cycle arrest caused by a DNA damage checkpoint can be detected by flow cytometry of DNA content [127,134] and antibody detection of Ser10-phosphorylated histone-H3, a widely recognised marker of mitosis [127,171]. Western blotting for Ser345-phosphorylated Chk1 can also be used to show that Chk1 is activated and therefore that the G2/M checkpoint was initiated [127].

The steps of overcoming cell cycle arrest and entering into mitosis with damaged DNA can be shown by light microscopy and time-lapse video microscopy of treated cell populations. Mitotic entry can also be detected by western blotting for key mitotic proteins such as cyclin B and Tyr15-phosphorylated Cdk1. Cells that are undergoing checkpoint adaptation can be assayed for mitotic entry by a Cdk1 activity assay that uses PP1-Cα as a recombinant Cdk1 substrate [172]. Entry into mitosis with damaged DNA can also be detected using immunofluorescence microscopy for both γH2AX and Ser10-phosphorylated histone H3, either separately or simultaneously. Finally, continuation with the cell cycle can be shown by light microscopy and time-lapse video microscopy where cells can be seen to divide in the presence of a DNA damaging agent. In addition to this the clonogenic assay can be performed using treated mitotic cells and this shows that cells are able to survive following treatment with a genotoxic agent (unpublished data) [173].

## 6. Conclusions

Although there are several different cell death pathways, the variety of cancer cell lines that have been shown to enter into mitosis after a genotoxic event suggests that mitotic entry is a major cellular response to damaged DNA. This may be because cancer cell lines are typically deficient in one or more proteins associated with the cell cycle and cell cycle checkpoints. We predict that many of these cell lines undergo checkpoint adaptation and that further research into DNA damage repair and the DDR will help to find links to the pathways that cells use to enter mitosis with damaged DNA. These studies are necessary to understand how cells arrive at certain fates, such as cell death or survival, after entering mitosis with damaged DNA. An improved understanding of these areas will provide a better insight into how current cancer therapies work and how they may fail, potentially leading to an improvement in their efficacy.

## Figures and Tables

**Table 1.** A table of different cancer treatments that damage DNA, their mechanism of action, and the main types of damage that they cause.

Agent Type	Examples of Drugs	Mechanism of Action	Type of DNA Damage
Alkylating agents	Nitrogen mustards Nitrosoureas Temozolomide S23906	Base alkylation-Monofunctional DNA adducts	Block the replication machinery leading to strand breaks
		Inter, intra and DNA-protein crosslinks	Block the replication machinery leading to strand breaks

Platinum drugs	Cisplatin Carboplatin Oxaliplatin	Monofunctional DNA adducts	Block the replication machinery leading to strand breaks
		Inter, intra and DNA-protein crosslinks	Block the replication machinery leading to strand breaks

Antimetabolites	5-Fluorouracil	Misincorporates into DNA	Blocks the replication machinery leading to strand breaks
		Depletes dNTPs	Blocks the replication machinery leading to strand breaks

Topoisomerase poisons	Camptothecin Etoposide	Inhibit topoisomerase enzymes in complex with DNA	SSBs and DSBs

Ionising radiation		Direct	SSBs and DSBs
		Indirect production of ROS	DNA adducts, base oxidation, SSBs, DSBs, base deamination, DNA-protein crosslinks

**Table 2.** A table listing the peak plasma concentration of clinical DNA damaging drugs, and their treatment schedule.

Drug	Administration	Dosage	Peak Plasma Concentration
Temozolomide	Oral capsule	200 mg/m^2^/day for 5 days	104 μM [45]
		150 mg/m^2^/day for 5 days	66 μM [45]
		100 mg/m^2^/day for 5 days	44 μM [45]

Cisplatin (Total platinum)		Toxic concentration in blood	16 μM [50]
	1 h intravenous	100 mg/m^2^	12 μM [51]
	Rapid (4–15 min) intravenous	100 mg/m^2^	40 μM [52]

Carboplatin (Total platinum)	1 h intravenous	400 mg/m^2^	70 μM [53]
	37 min intravenous	350 mg/m^2^	130 μM [54]

Oxaliplatin (Total platinum)	5 cycles, 2 h intravenous	130 mg/m^2^ every 3 weeks	10 μM [55]
	3 cycles, 2 h intravenous	85 mg/m^2^ every 2 weeks	6 μM [55]

5-Fluorouracil	72 h intravenous	1750 mg/m^2^/day	10 μM [56]

Camptothecin	Oral	1.5 mg/m^2^/day for 5 days	75 nM [57]

Etoposide	Oral	100 mg/day-8–15 days (typical dose)	14 μM [58]
	Intravenous, 500 mg/h	400–800 mg/m^2^/day for 3 days (high dose study)	45–194 μM [59]

## Abbreviations

ATM: Ataxia telangiectasia mutated
ATR: ATM and Rad3-related
ATRIP: ATR-interacting protein
Cdk1: Cyclin-dependent kinase 1
Cdk2: Cyclin-dependent kinase 2
Chk1: Checkpoint kinase 1
Chk2: Checkpoint kinase 2
CPT: Camptothecin
DDR: DNA damage response
DNA: Deoxyribonucleic acid
dNTPs: Deoxynucleotide triphosphates
DSBs: Double-strand breaks
hmdUrd: 5-hydroxymethyl-2′-deoxyuridine
MGMT: *O*^6^-methylguanine-DNA methyltransferase
MRN: Mre11-Rad50-Nbs1
RFC: Replication factor C
ROS: Reactive oxygen species
RPA: Replication protein A
SSBs: Single-strand breaks
ssDNA: Single-stranded DNA
TMZ: Temozolomide
TopB1: DNA topoisomerase II binding protein 1
Top1: Topoisomerase I
Top2: Topoisomerase II
UV: Ultraviolet
5-FU: 5-fluorouracil
9-1-1: Rad9-Rad1-Hus1
